# Secondary Analysis of Human Bulk RNA-Seq Dataset Suggests Potential Mechanisms for Letrozole Resistance in Estrogen-Positive (ER+) Breast Cancer

**DOI:** 10.3390/cimb46070424

**Published:** 2024-07-06

**Authors:** Lincoln Sutherland, Jacob Lang, Norberto Gonzalez-Juarbe, Brett E. Pickett

**Affiliations:** 1 Department of Microbiology and Molecular Biology, Brigham Young University, Provo, UT 84602, USA; suthe8@student.byu.edu (L.S.); jlang18@student.byu.edu (J.L.); 2 J. Craig Venter Institute, Rockville, MD 20850, USA; ngj@umd.edu; 3 Department of Cell Biology and Molecular Genetics, University of Maryland, College Park, MD 20742, USA

**Keywords:** estrogen receptor, breast cancer, treatment resistance, letrozole, transcriptional mechanisms

## Abstract

Estrogen receptor-positive (ER+) breast cancer is common among postmenopausal women and is frequently treated with Letrozole, which inhibits aromatase from synthesizing estrogen from androgens. Decreased estrogen slows the growth of tumors and can be an effective treatment. The increase in Letrozole resistance poses a unique problem for patients. To better understand the underlying molecular mechanism(s) of Letrozole resistance, we reanalyzed transcriptomic data by comparing individuals who responded to Letrozole therapy (responders) to those who were resistant to treatment (non-responders). We identified *SOX11* and *S100A9* as two significant differentially expressed genes (DEGs) between these patient cohorts, with “PLK1 signaling events” being the most significant signaling pathway. We also identified *PRDX4* and *E2F8* gene products as being the top mechanistic transcriptional markers for ER+ treatment resistance. Many of the significant DEGs that we identified play a known role in ER+ breast cancer or other types of cancer, which partially validate our results. Several of the gene products we identified are novel in the context of ER+ breast cancer. Many of the genes that we identified warrant further research to elucidate the more specific molecular mechanisms of Letrozole resistance in this patient population and could potentially be used as prognostic markers with further wet lab validation. We anticipate that these findings could contribute to improved detection and therapeutic outcomes in aromatase-resistant ER+ breast cancer patients.

## 1. Introduction

Breast cancer is the second leading cause of cancer death in women [1]. Approximately 13% of women will develop invasive breast cancer, with 3% of women dying from it [1]. Roughly 83% of invasive breast cancer cases are diagnosed in post-menopausal women (50 years or older), with 91% of the deaths occurring in the same age group [1,2]. Given that those who are 65 years of age or older are the largest growing subpopulation in the United States (16.8% of the total population in 2020) [3], the overall number of new breast cancer cases is expected to rise accordingly. The American Cancer Society predicts that over 310,000 women in the United States will be diagnosed with breast cancer in 2024 [4], which is expected to rise to ~440,000 by 2030 [5].

Breast cancer can be classified as a heterogenous disease. Among the various types of breast cancer, some types have an affinity for particular hormones that contribute to rapid tumor growth. Three important markers that are generally used to classify breast cancer involve the positive or negative presence of the estrogen receptor (ER), progesterone receptor (PR), and/or the human epidermal growth factor receptor 2 (HER2) protein on the cell surface [6]. The estrogen and progesterone receptors both belong to the steroid receptor subgroup of ligand-activated transcription factors within the nuclear receptor superfamily [7]. Their presence is a strong predictor of the efficacy of adjuvant and therapeutic hormonal therapy [7]. Amplification of the *ERBB2* gene in tumor cells leads to increased presence of the protein at the cellular membrane, leading to potent proliferative and anti-cell death signaling [8,9]. Taken together, the ER, PR, and HER2 play a central role in the detection, diagnosis, and therapy of breast cancer [10,11,12,13].

Given that estrogen contributes to the promotion and progression of breast cancer, some therapeutic strategies specifically target estrogen synthesis via the ER and its associated intracellular signaling pathways [14]. The aromatase enzyme then catalyzes the final conversion of androgens into estrogens [14,15]. Thus, aromatase was identified as a potential therapeutic target in the late 1970s with the synthesis of aminoglutethimide as the first aromatase inhibitor (AI) [16]. The low specificity and selectivity of this early therapeutic led to the synthesis of subsequent aromatase inhibitors, with the most recent being Letrozole [16]. The increased affinity of Letrozole to the catalytic site of the aromatase enzyme competitively inhibits the binding of the precursor reactant to the aromatase enzyme [14]. Moreover, Letrozole has greater potency than other Ais, including anastrozole, exemestane, formestane, and aminoglutethimide [14]. Seventy-seven percent of all breast cancers are ER-positive (ER+); thus, treatment with aromatase inhibitors is currently the standard treatment for all postmenopausal women [7]. Although Letrozole is one of the most commonly used treatments for ER+ breast cancer, it also has limitations [17,18]. A prior study showed that the overall response rate (ORR) among a large multi-national study of postmenopausal women was 55% [17]. The ORR quantitatively represents the percentage of patients that achieve a favorable response, either total (complete disappearance of cancerous tissue) or partial (defined as a decrease in tumor size by at least 30%) [19]. Response rates are determined by either a mammogram or an ultrasound [17]. To our knowledge, the ability of existing prognostic methods to accurately identify treatment resistance prior to treatment is lacking.

A prior study examining the intracellular transcriptional response to Letrozole treatment used RNA-sequencing data to identify Proline-Rich Protein 11 (*PRR11*) as the only significantly overexpressed gene among 51 genes in chromosome arm 17q23. Focusing their analysis and validation work on this single PRR11-containing locus in treatment-sensitive vs. treatment-resistant ER+ breast cancer patients was useful [20], and amplification of this locus has been shown to correlate with poor clinical outcomes in breast cancer [21].

The aim of the current study is to perform a secondary analysis of the complete transcriptome from the same original RNA-sequencing study in treatment-sensitive vs. treatment-resistant ER+ breast cancer patients. We expect that re-analyzing this valuable dataset will identify genes, signaling pathways, and transcriptional mechanisms across the whole human transcriptome that are significantly associated with resistance to letrozole treatment in patients with ER+ cancer.

## 2. Materials and Methods

### 2.1. Retrieving Fastq Files, Preprocessing, and Enriching RNA-Sequencing Data

The sratools software (version 2.10.8) package was used to download the RNA-sequencing fastq files from the desired study (Gene Expression identifier: GSE145325) from the Sequence Read Archive (SRA) database at NCBI [20,22]. The fastq files were then pre-processed and analyzed using the snakemake-based Automated Reproducible Modular Workflow for Preprocessing and Differential Analysis of RNA-seq Data (ARMOR; version 1.5.7) [23,24]. This preprocessing workflow applies the following methods using established algorithms: (1) quality control with fastqc [25] using default parameters; (2) trimming with TrimGalore! [26] using default parameters, including a minimum quality score of 20, a phred scale of 33, and a minimum length of 20 bases; (3) pseudomapping and quantification to the human transcriptome (GRCh38 release 98) using Salmon (version 1.3.0) [27] with a k-mer setting of 31, a read length of 63, and the following flags: seqBias,gcBias, fldMean=250, and fldSD=25; (4) read count normalization and differential gene expression using edgeR (version 3.36.0) [27,28] with default parameters; and (5) functional enrichment using CAMERA (version 3.50.3) [29], with default parameter enriching against the H, C2, and C5 Molecular Signature database gene sets. Although the trimming and/or differential expression algorithms may change, each of these steps is commonly applied to preprocessing RNA-sequencing data.

The differential gene expression results were then used as an input to the Signaling Pathway Impact Analysis (SPIA; version 2.50.0) algorithm [30], with default parameters other than using 2000 replicates and a Bonferroni-adjusted *p*-value cutoff of 0.05. This algorithm uses bootstrapping and a null distribution to calculate significantly perturbed pathways that are enriched in statistically significant DEGs, regardless of the fold-change value or direction [31,32,33]. The SPIA method was specifically chosen because of its ability to take into account the topology of gene products that contribute to a pathway rather than simply calculating whether there is a significant overlap between the significant genes and the pathway, which decreases the number of false-positive results. Another benefit is that this algorithm uses bootstrapping and permutation to calculate a custom null distribution for each pathway. This null distribution is then used to determine a specific and unique *p*-value for the differentially expressed genes in the dataset for each pathway. This approach generally produces fewer false-positive results when compared to other hypergeometric-based methods and enables it to predict whether any significant pathway is activated or inhibited without needing to perform separate analyses of upregulated and downregulated DEGs [30,31,32,33].

### 2.2. Target and Mechanistic Marker Prediction

The Pathway2Targets algorithm (version 2.2) was then used to predict and prioritize relevant therapeutic targets from the transcriptomics signatures that could be repurposed in the case of Letrozole resistance, similar to prior work [34,35,36,37]. Briefly, this algorithm identifies known drug targets within each of the significant signaling pathways. It then uses multiple criteria from the OpenTargets database [38], as well as the signaling pathway information, to rank the relevant therapeutics and their targets according to a customizable weighting scheme. The justification for this approach is that a gene product does not need to be differentially expressed to be a relevant therapeutic target that is capable of reducing the signs and/or symptoms of a given condition, particularly if the target is upstream of one or more significant DEGs.

Separately, the Salmon read counts for each sample were combined into a tabular format and labeled as “resistant” or “sensitive” to treatment with Letrozole. This table was then used as an input to the XGboost algorithm [39], which implements a tree-based method to train a model from 80% of the dataset and then quantifies its performance using the remaining 20% of the data [40,41], which minimizes model overfit. For the initial analysis, the gain metric was calculated from the read counts for all detected genes across all samples. The results were sorted in descending order, such that the genes/features with the highest gain value were listed first. Given the gain metrics from the whole transcriptome, the number of genes/features being evaluated was reduced to the best two mechanistic markers from the original analysis since this is a number that is easily accommodated by qRT-PCR (or similar) molecular methods. This approach has been successfully applied previously with acceptable performance and accuracy [40,41,42]. The XGboost algorithm was selected since prior work has shown that tree-based classifiers are faster and more accurate than other machine learning-based methods such as support vector machines, neural networks, and Bayesian approaches [43]. The XGboost parameters that were changed from the default included: constructing 10,000 parallel trees to ensure appropriate coverage of the available tree space, a subsample of 0.5, using a binary–logistic objective, and an area under the curve (AUC) evaluation metric.

### 2.3. Protein Network Analysis

The public protein–protein interactions in the STRING database were used to construct a PPI network for all significant DEGs, the top five targets, and the top five mechanistic transcriptional markers [44]. Cytoscape was then used to visualize the network, while the 12 algorithms incorporated into the cytoHubba app were used to compute multiple node-based metrics from the Cytoscape network [45,46]. These algorithms were specifically selected to predict key nodes from a network while also minimizing bias due to either high-degree or low-degree proteins [46].

## 3. Results

### 3.1. The Majority of Differentially Expressed Genes in ER+ Treatment Resistance Are Upregulated

We began by retrieving, preprocessing, and analyzing an existing dataset, which consisted of 58 ER+ stage II or stage III primary breast tumor samples from newly diagnosed female patients who had been treated with Letrozole for a median of 7.2 months prior to surgery. These patients were at least 60 years old when the original study started, had a mix of progesterone receptor presence, were primarily HER2-negative, and consisted mostly of ductal carcinoma. No mention of prior treatment with other therapeutics was associated with the original study.

The RNA sequencing data from these clinical samples are publicly available in the NCBI Gene Expression Omnibus (GEO) database. Our analysis detected a total of 18,735 genes between responders (treatment-sensitive patients) and non-responders (treatment-resistant patients), with 105 of those genes having significant differential expression (FDR *p*-value < 0.05; Table 1; Supplementary Table S1). This significance cutoff was used since the FDR method applies a multiple hypothesis correction to reduce false positives in the results when many statistical tests are performed.

**Figure 1:**
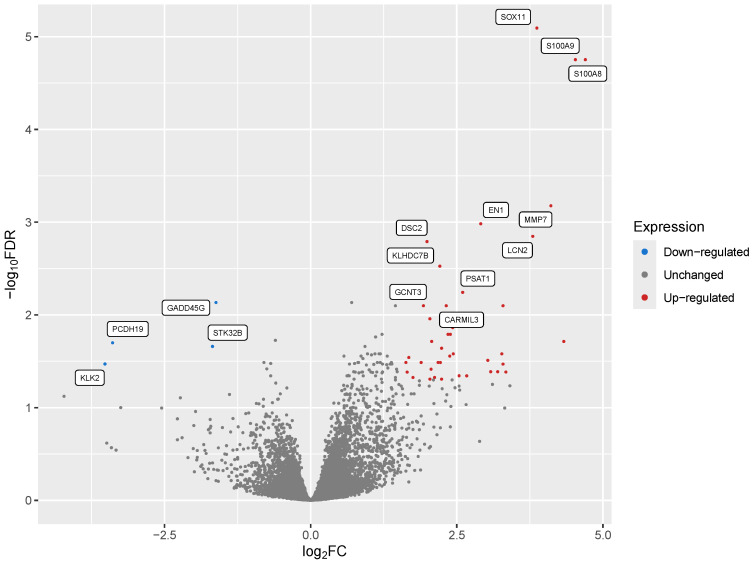
A volcano plot of all differentially expressed genes (DEGs). Each dot represents a gene product, with blue dots representing those that were significantly downregulated (log_2_FC < 1; FDR-corrected *p*-values < 0.05) and red representing DEGs that were significantly upregulated (log_2_FC > 1.5; FDR-corrected *p*-value < 0.05, equivalent to −log_10_FDR of 1.3). The 15 most statistically significant DEGs (11 up-regulated and 4 down-regulated) are labeled.

We observed that among these 105 Differentially Expressed Genes (DEGs) (Figure 1), the top 20 (Table 2) included 17 protein-coding genes that consisted of two S100 calcium-binding proteins, various transcription factors, and matrix metallopeptidase 7 (Figure 1 and Table 2). Three of the top twenty DEGs coded for immunoglobulin variable regions, while one was a putatively transcribed unprocessed pseudogene, Ovostatin II. All but one of these 20 DEGs were upregulated in the group that failed to respond to Letrozole treatment. The sole exception to this was the downregulation of the gene that encodes the growth arrest and DNA-damage inducible gene 45 gamma expression (GADD45G), which is involved in the regulation of cell growth and apoptosis [47] and showed a log2 fold-change (log2FC) value of −1.62.

The original study identified *PRR11* as one of the few statistically significant differentially expressed genes among the 51 genes in chromosome arm 17q23 that were included and validated in their analysis [20]. However, our analysis identified *PRR11* to be the 1211th differentially expressed gene across the whole transcriptome, which did not surpass the statistical (FDR-adjusted *p*-value < 0.05) threshold for significance.

### 3.2. The Signaling Pathway Impact Analysis Identified Four Significantly Affected Pathways

We then wanted to determine which intracellular signaling pathways were significantly enriched with the detected DEGs using the Signaling Pathway Impact Analysis (SPIA) algorithm (Bonferroni-adjusted *p*-value < 0.05). Briefly, this robust pathway enrichment algorithm uses a bootstrap-based approach to generate a null distribution for each pathway and calculate a *p*-value. The results from our pathway enrichment analysis included four pathways that were predicted to differ between responders and non-responders to Letrozole treatment (Table 3). These pathways included those associated with activated “PLK1 signaling events and anti-tumoral activity” as well as the activated “FOXM1 proliferation-associated transcription factor network”.

### 3.3. Targets Prioritized for Repurposing from the Identified Pathways

We then wanted to use the pathway results to predict any existing therapeutic targets that could be repurposed to treat the resistance phenotype with small molecules, monoclonal antibodies, peptides, or other modalities. We consequently applied the Pathway2Targets algorithm to perform this target prioritization analysis, which predicted 60 therapeutic targets (Table 4 and Supplementary Table S2). Briefly, this algorithm calculates a weighted score for each target based on the sum of values assigned to 26 attributes. Notably, the predicted targets for the non-response (resistance) phenotype included Vascular endothelial growth factor A (VEGFA), a current target for solid tumors, as well as Estrogen Receptor 1 (ESR1), Nitric Oxide Synthase 2 (NOS2), and various matrix metalloproteinases (MMP9 and MMP2).

### 3.4. Machine Learning Predicted Two Robust Mechanistic Transcriptional Markers

**Figure 2:**
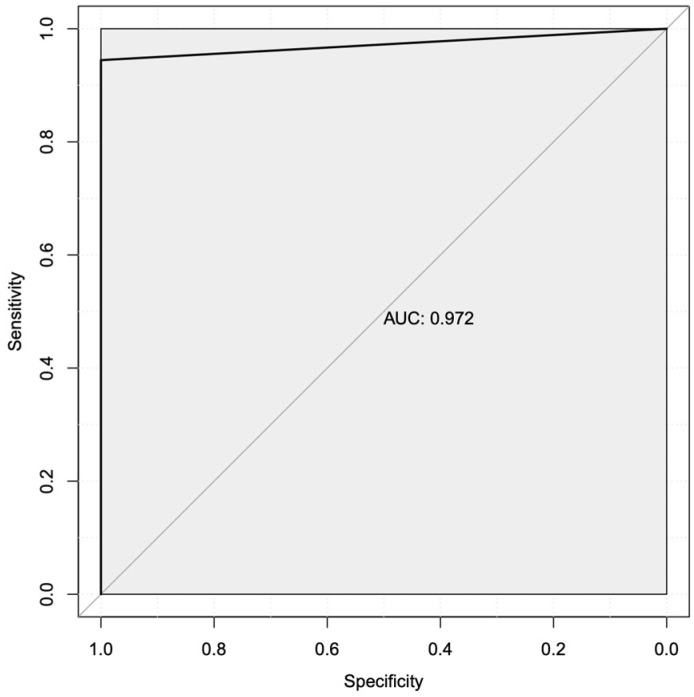
The area under the receiver operator characteristic (AUROC) curve shows high predicted sensitivity vs. specificity using all transcripts. An initial machine learning analysis using all detected gene products as inputs showed high overall specificity (*x*-axis) and sensitivity (*y*-axis) for detecting Letrozole resistance in patients, with an AUC value of 97.2%.

We next wanted to predict a subset of mechanistic transcriptional markers that could potentially be used to better identify patients who will not respond to treatment with Letrozole and better understand their underlying resistance mechanism(s). To perform this analysis, we applied a decision tree-based machine learning approach to predict features (i.e., expressed genes) that most accurately classify Letrozole treatment responders vs. non-responders. We used the read counts for all samples as the input for this analysis to identify and rank 278 transcriptional biomarkers by the gain metric (Table 5). The performance metrics for all 278 transcriptional biomarkers yielded an area under the receiver-operator characteristic (AUROC) curve of 0.972 (97.2%; Figure 2), indicating an exceptional ability to classify Letrozole responders vs. non-responders based on global gene expression. The sensitivity for this complete set of mechanistic transcriptional markers was predicted to be 100%, with a predicted specificity of 94%.

**Figure 3:**
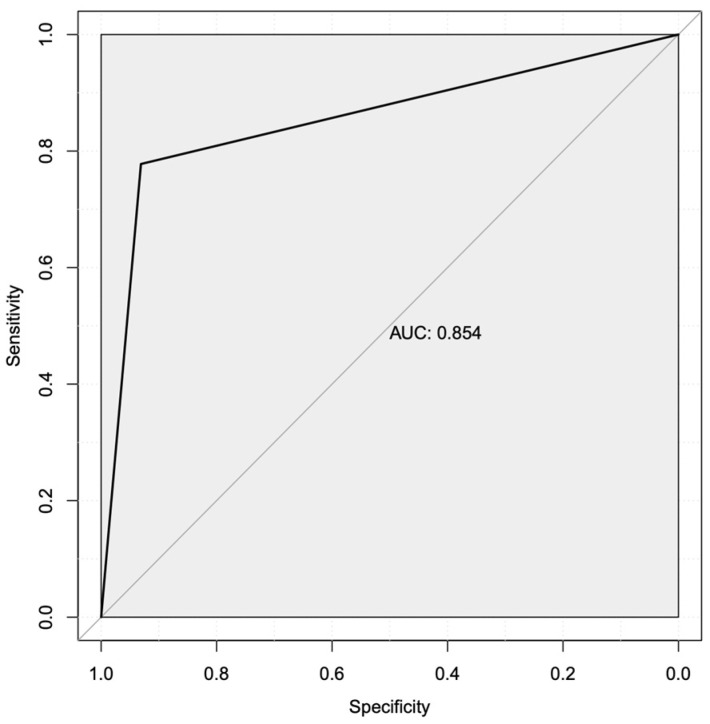
The area under the receiver operator characteristic curve shows acceptable performance for the top two transcripts. A secondary computational analysis using the highest ranked mechanistic markers from the whole transcriptome prediction (PRDX4 and E2F8). This analysis showed the combination of these two gene products having acceptable specificity (*x*-axis) and sensitivity (*y*-axis) for detecting Letrozole resistance in patients, with an AUC value of 85.4%.

Given the resources required to generate whole transcriptome data using RNA-sequencing, we repeated our machine learning analysis to predict the mechanistic transcriptional markers that are most associated with treatment nonresponse in this patient population. To achieve this, we used the top two mechanistic markers from our first analysis as an input for a subsequent analysis. This more focused second analysis identified PRDX4 and E2F8 transcripts as markers with potential as diagnostic targets, with an AUROC for only these two gene products of 0.854 and an overall accuracy of 88.2% (Figure 3).

### 3.5. Protein–Protein Interactions Reveal a Potential Treatment Resistance Network

**Figure 4:**
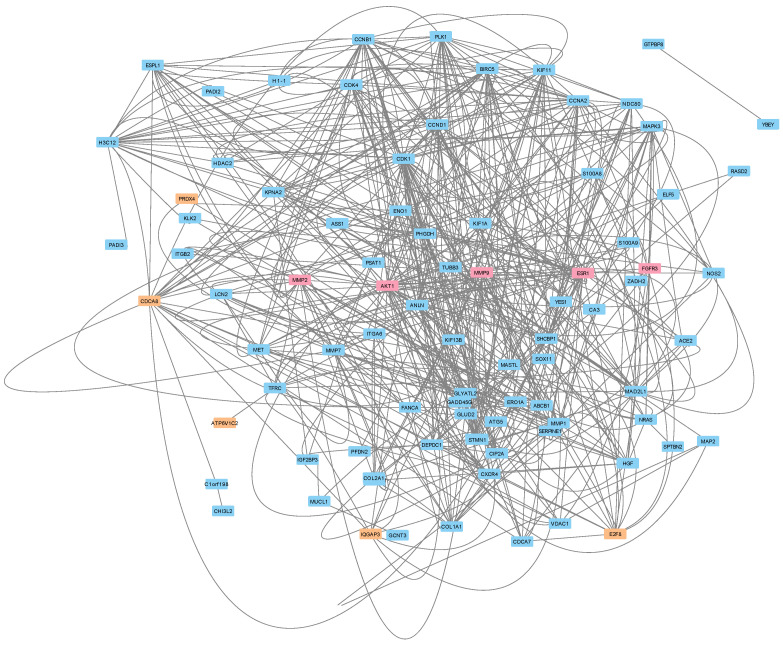
Protein–protein interaction network of DEGs, predicted targets, and top predicted markers. A graphical representation showing the interactions (edges) for each of the proteins (nodes) from the STRING-DB database. The 87 gene products that directly interacted with each other were included in a single network. The input consisted of the 105 DEGs (blue-colored nodes), top five predicted therapeutic targets (salmon-colored nodes), and top five predicted transcriptional biomarkers (orange-colored nodes).

We then constructed a protein–protein interaction (PPI) network to predict the protein(s) that potentially played a role in Letrozole resistance for these patients (Figure 4). To achieve this, we retrieved the known PPIs from the public STRING database for the 105 DEGs, as well as the top five drug targets and the top five transcriptional biomarkers. Interestingly, this analysis revealed a relatively well-connected local network, which indicates that the majority (87/115) of the input gene products directly interacted with each other and at least partially supports our earlier predictions. We applied all 12 available algorithms within the CytoHubba app to this network to identify the key proteins with minimal bias to any individual approach. We then calculated the rank determined by each algorithm and then averaged them together. This analysis identified the top 10 “key” proteins in the network as CDK1, CCNB1, CCNA2, BIRC5, AKT1, ESR1, MMP9, CDCA8, CCND1, and PLK1. We found the ESR1, PLK1, MMP9, and AKT1 proteins to be of particular interest since they were identified previously as the targets of Letrozole, a significant signaling pathway, and two therapeutic targets. This profile at least partially represents the intracellular transcriptional response to Letrozole therapy.

## 4. Discussion

The primary aim of the current study was to perform a secondary analysis of a publicly available RNAseq dataset produced from 58 samples from patients with ER+ breast cancer following Letrozole treatment. In addition to finding PRR11-related mechanisms, the original study reported a cluster of E2F4-regulated genes that were sensitive to subsequent CDK4/6 inhibitor (palbociclib) treatment in a separate cohort of ER+ breast cancer patients [48].

Our analysis identified 105 statistically significant DEGs, with the top 20 gene products consisting of 17 protein-coding genes, including three encoding immunoglobulin variable regions and one pseudogene. We also identified four significant signaling pathways, twenty therapeutic targets that could be repurposed to reduce Letrozole resistance and/or target resistance cells, and two potential mechanistic transcriptional markers that demonstrate high combined specificity and sensitivity in identifying Letrozole non-responders. Below, we describe our primary findings in the context of breast, and other cancer-related studies in an effort to use prior observations to at least partially validate our mechanistic findings.

### 4.1. Differentially Expressed Genes

In contrast to our findings, which identified 105 statistically significant DEGs, the original study that utilized the same data identified PRR11 as the only differentially expressed gene among the 51 genes in chromosome arm 17q23 [20]. We believe that the mostly likely contributor to this seeming discrepancy was the focus of the original study on the particular locus in 17q23 that contains 51 genes. In contrast, our analysis evaluated all of the detected mRNAs in the cells, which increased the scope and the statistical stringency needed to obtain significant results after incorporating multiple hypothesis corrections. PRR11 underwent substantial differential gene expression in our analysis, although our thresholds did not categorize it as significant when the whole transcriptome was taken into account.

Transcription of the SRY-Box Transcription Factor 11 (*SOX11*) gene (with a log2FC value of 3.87) was our most statistically significant finding. Briefly, *SOX11* is part of the Sox gene family, comprising 20 transcription factors, which can further be divided into nine subgroups based on functional similarity [49]. *SOX11* belongs to the SoxC group (composed of Sox4, Sox11, and Sox12), which are critical in the development of the nervous system and show substantial transactivation potential [50], while also being required for the generation/proliferation of immature neuron progenitors [51]. *SOX11* has recently been shown to behave as an oncogene and is a critical regulator of basal-like and luminal B breast cancers and their metastasis to the brain [52,53,54].

The upregulated S100 calcium-binding Protein A8 and A9 (*S100A8* and *S100A9*) genes encode a heterodimer of two calcium-binding proteins, referred to as calprotectin [55]. This protein is secreted by neutrophils and is an important proinflammatory mediator in both chronic and acute inflammation [55]. Calprotectin has recently been discovered to play an important role in tumorigenesis, cell proliferation, and resistance to traditional cancer therapies [56,57]. S100 proteins lack the signal peptide required for secretion via the classical endoplasmic reticulum/Golgi route, and its secretion mechanism is not well understood [56,58]. Recent discoveries implicate BRCA1, a known tumor suppressor, as essential for regulating the levels of S100A8 and S100A9 in the body [56,59]. These S100 gene products have also been shown to be secreted due to IL-22 and IL-17 signaling [60], have been associated with poor outcomes in ductal carcinomas [61], and have been affected after AI treatment in an animal model [62]. While S100 proteins have been shown to be host markers of tumor development and progression, their role as modulators of Letrozole resistance has not been characterized previously. Thus, we believe that further experiments will help to elucidate the underlying mechanism(s) of S100A8 and S100A9 proteins in treatment-resistant ER+ breast cancer.

The Immunoglobulin Lambda Variable 3–25 (*IGLV3–25*) gene codes for light chain variable regions involved with antigen recognition [63,64] and has previously been implicated in various cancers [63,65,66]. Our analysis also found Matrix Metalloproteinase 7 (*MMP7*) to be upregulated in this dataset. This gene product encodes a proteolytic enzyme that secretes zinc and calcium endopeptidases, which are normally involved in wound healing and bone growth [67]. It has also been implicated in cancer progression, proliferation, differentiation, and/or apoptosis [68,69,70]. Basal-like breast cancer has upregulated *MMP7* expression, which has been linked to *DKK1* knockdown, a known tumor suppressor in breast cancer [71]. Interestingly, zoledronate, a common drug used to treat bone damage and osteoporosis [72,73,74], has been shown to decrease the expression of *MMP7* in breast cancer cell lines [75].

Engrailed Homeobox 1 (*EN1*) was another upregulated gene that we identified, which is a protein-coding gene that helps control development [76] and has been shown to affect bone metastases from breast cancer tumors [77]. It has been implicated in quadruple-negative breast cancer and basal-like breast cancer [78,79,80]. Our findings could potentially expand its scope of relevance to treatment-resistant ER+ breast cancer. We believe that future validation experiments should be performed to confirm these differential expression results.

### 4.2. Intracellular Signaling Pathways

Our analysis found four notable intracellular pathways that were significantly modified among non-responders. This analysis predicted the “PLK1 signaling events”, “Syndecan-1 mediated signaling events”, and “FOXM1 proliferation transcription factor network” pathways to be activated; the “HIF-1-alpha transcription factor network” was predicted to be inhibited. Although the number of pathways is relatively small compared to other algorithms, we believe that applying a Bonferroni *p*-value correction minimized the number of false results. It is possible that this approach could be complemented by other approaches in future studies.

PLK1-related signaling was our most significantly impacted intracellular signaling pathway (activated among non-responders vs. responders) and is primarily involved in cell mitosis. PLK1 contributes to many functions, including controlling the G2/M checkpoint, coordinating the cell cycle, and serving as a regulator of cell division in eukaryotes, as well as in both DNA replication and chromosome segregation [81,82]. In general, the overexpression of PLK1 has been associated with various cancer types, including ER+ breast cancer [82,83]. PLK1 inhibition has been shown to exhibit anti-tumoral activity by blocking mitosis in cells with higher PLK1 expression, making it an attractive target for cancer therapy [84,85]. PLK1 knockdown in hormone-independent ER+ breast cancer has been associated with decreased cell viability and sensitization to radiation treatment [86]. These past studies serve to partially validate our significant pathway findings and lend credence to ongoing research into PLK1 inhibition as a potential treatment for patients with ER+ breast cancer.

The pathway for Syndecan-1 (SDC1, CD138) signaling was activated in non-responders. SDC1 is a transmembrane heparan sulfate proteoglycan responsible for maintaining typical cell morphology and is found abundantly in epithelial cells [87]. The SDC1 pathway is generally involved in cell proliferation, cell–matrix interactions, growth factor signaling, and angiogenesis [88,89]. SDC1 overexpression tends to correlate with more aggressive tumors, and it has an inverse correlation with ER expression [90]. This inverse correlation between ER expression and SDC1 signaling could help explain why Letrozole was ineffective for at least a subset of non-responders and merits further experimentation. Our analysis also showed MMP-7 as a highly significant DEG, which has been shown to anchor itself to syndecans and results in the shedding of important membrane-bound ligands, such as EGF and TGF-a, leading to cell invasion [69]. Future follow-up experiments to validate these findings could include using CRISPR-i (or similar) systems and/or drugs to affect these pathways.

### 4.3. Target Prioritization and Repurposing

Our approach to predicting potential therapeutic targets by combining public data with analysis-specific pathway information and a customizable weighted scoring method is unique. While this approach has been useful in the past [32,33], other approaches exist that could complement our findings. Specifically, since the target data comprise more antagonists than agonists, this could potentially be a limitation. Vascular endothelial growth factor A (VEGFA) was the top potential target identified by our prioritization algorithm, which has already been approved for certain indications, including breast cancer. This growth factor belongs to a ligand family composed of six related proteins and plays an important role in breast cancer and other forms of cancer [91]. The primary role of this protein is to stimulate angiogenesis, which facilitates tumor growth [91,92]. Our computational prediction of VEGFA as a relevant target warrants future experimentation and serves as at least a partial validation of our approach.

Estrogen Receptor 1 (ESR1) encodes the estrogen receptor and was identified as a therapeutic target. Of note, ESR1 has been found to encode gain-of-function mutations that promote tumor metastasis and resistance to endocrine therapy [93,94,95] and can be detected with a liquid biopsy [94]. The detection of ESR1 as a therapeutic target is logical given that resistance to AI treatment enables the continued activation of signaling pathways that are modulated by ESR1.

Two additional highly ranked targets identified by our algorithm were Matrix Metallopeptidase 9 (MMP9) and Matrix Metallopeptidase 2 (MMP2), both members of the extracellular matrix metalloproteinase family (MMPs). MMPs assist in extracellular matrix remodeling through the activation of substrates via enzymatic cleavage [96]. They are involved in a variety of normal processes in the body, and their dysregulation plays a vital role in processes such as aging. Their dysregulation also plays a role in several abnormal conditions, including preeclampsia among pregnant women and breast cancer [96,97,98]. Higher levels of MMP9 have been correlated with higher tumor grades and resistance to endocrine therapy [96]. MMP9 was further characterized as a vital component of the metastatic cascade early in tumorigenesis that promotes colonization in the lungs [99]. Other studies have implicated MMP2 with tumor metastasis to other organs, including the brain [100,101]. Overall, both MMP9 and MMP2 have been identified as promising markers for predicting the prognosis of patients with breast cancer [102,103]. Interestingly, a phase III clinical trial targeting MMP proteins with marimastat sought to reduce tumor blood flow in patients with metastatic breast cancer [104]. Although MMP7 was identified as a significant DEG, it was not predicted as a useful target in this analysis. However, MMP7 is one of the several MMPs that are inhibited by doxycycline, which is a commonly used treatment for various forms of cancer.

Fibroblast growth receptor 3 (FGFR3) belongs to a family of four highly conserved, transmembrane receptor tyrosine kinases. Once activated, they initiate intracellular cascades that carry out a variety of functions, including promoting cellular proliferation and survival [105]. This gene has been reported in other studies to be significantly upregulated in breast cancer [106]. FGFR3 is a known target of microRNA-593-3p, and the overexpression of miR-593-3p down-regulates FGFR3 expression, which slows breast cancer progression [107]. Various inhibitors of FGFR3 are at various stages in phase I and phase II clinical trials for breast cancer.

Another potential target we identified was AKT Serine/Threonine Kinase 1 (AKT1). AKT1, AKT2, and AKT3 are all isoforms of protein kinase B (AKT), an essential member of the PI3K/AKT signaling pathway [108]. Both AKT1 and AKT2 have been implicated in breast cancer previously, with some mixed results regarding their specific function. However, it is generally accepted that AKT1 plays an important role in the early stages of tumor development (tumor initiation and proliferation), while AKT2 primarily assists in tumor metastasis [108,109]. The original study for this dataset identified PI3K as an important player in AI treatment [20], which is supported in other studies [110]. Regardless of its specific function, AKT1 has a well-documented correlation with breast cancer and is an appropriate target for pharmaceutical intervention. Additional work at the discovery, pre-clinical, and clinical stages is needed to determine whether such treatments could reduce the effect of Letrozole resistance in breast cancer patients.

### 4.4. Mechanistic Transcriptional Marker Analysis

Our DEG analysis identified expressed genes that significantly differed between the two groups of samples (resistant vs. sensitive). In contrast, the aim of our mechanistic marker analysis was to determine the expressed genes that most consistently differed between the two groups of samples. Although the input for both analyses was the read counts, we employed a tree-based machine learning model for the mechanistic marker analysis that does not incorporate traditional statistics. Consequently, we did not expect a complete overlap between the DEGs and our predicted biomarkers.

Peroxiredoxin-4 (*PRDX4*), which was an up-regulated gene among non-responders, was the top transcriptional mechanistic marker identified in our analysis. This gene product belongs to a family of six small antioxidant isozymes and is generally localized in the endoplasmic reticulum [111]. PRDX4 plays an essential role in maintaining localized redox homeostasis and is upregulated when cells are under oxidative stress [112]. PRDX4 has been described as having a tumor-promoting effect that is well-documented among various forms of cancer, including lung and renal cancer, leukemia, and glioblastoma [113,114]. Prior work has shown the overexpression of PRDX4 to be associated with poor overall survival rates, shorter relapse-free survival among breast cancer patients [115,116], and metastasis. PRDX4 has also been shown to be associated with more advanced breast cancer tumors [117], which could also contribute to treatment resistance. Interestingly, PRDX4 was also identified as a potential therapeutic target for prostate cancer, suggesting a possible shared mechanism for androgens in both prostate and breast tissues [118,119]. Thus, the role of PRDX4 as a potential mechanistic marker and potential target in treatment-resistant breast cancer deserves future exploration.

Early Region 2 Binding Factor (*E2F8*), an up-regulated DEG from our study, was another potential mechanistic marker identified by our machine learning analysis. *E2F8* is a member of the E2F family, which assists in assembling the core transcriptional machinery, making it crucial for cell division [120]. E2F8 has also been shown to promote angiogenesis, which helps establish the tumor microenvironment and is positively correlated with tumor malignancies [121,122]. E2F8 has been associated with CDK4/6 inhibitor resistance [123], is overexpressed in patients with breast cancer, and has been significantly correlated with poor patient survival and cancer progression [124,125]. E2F8 has been suggested as a marker of breast relapse-free survival and distant metastasis-free survival in breast cancer patients [126]. Interestingly, a prior study in HER2- has identified a different E2F Transcription Factor (E2F4) as having a role in treatment resistance in ER+ breast cancer [127] and also in the original analysis of our same ER+ cohort [48]. As such, we believe that the role of E2F8 as a mechanistic marker, specifically in aromatase-resistant breast cancer, warrants further exploration.

We acknowledge inherent deficiencies in training and applying a machine learning approach to a relatively small dataset. Unfortunately, although the GEO database had other bulk RNA-sequencing datasets that characterized HER2+ samples, there were no other public ER+ Letrozole-treated clinical samples. Consequently, augmenting our model with additional data was not possible. Additional wet lab validation experiments using qRT-PCR or similar transcriptional assays will be required in one or more distinct patient populations to better quantify the accuracy and performance of our predicted mechanistic markers.

### 4.5. Potential Treatment Resistance Mechanisms

Unfortunately, the original dataset only included samples from responsive and non-responsive tumors. As such, it was not possible to directly and accurately analyze and assess the pre-treatment state of the tumor. Similarly, we believe it would not be justified to incorporate additional comparisons from other datasets that evaluated triple-negative or ER- breast cancers given the distinct patient populations, enrollment criteria, and other experimental variables. Even so, our protein network analysis did identify a set of gene products that may contribute to an increased understanding of the intracellular transcriptional response to Letrozole treatment.

We recognize that a subset of the genes we identified in our analysis of this dataset are opposite of what has been reported in prior work, specifically MMP9 and ESR1, as being upregulated in Letrozole-resistant breast cancer tumors, which agrees with our findings [128]. Another study found that estrogen is known to attract and activate neutrophils, which consequently upregulates the expression of both S100A8 and S100A9, in agreement with our findings [129]. Multiple immunoglobulin lambda genes, which were upregulated in our analysis, agree with prior work in humans and mice that showed a similar response in resistant samples [130,131]. SOX11 has been reported to be downregulated due to epigenetic effects in some breast cancer tumors after treatment with Letrozole [52]; however, SOX11 was found to be upregulated in our analysis. The fact that SOX11 was included in our protein network suggests that changes in its bulk RNA-seq expression could be driven by Letrozole treatment, though we are unsure whether this upregulation primarily takes place within tumor cells or in the tumor-associated immune cells [129]. We anticipate that additional experiments in cell culture and/or clinical cohorts will be necessary to validate these findings.

### 4.6. Study Limitations

We are aware that our analysis was not able to incorporate at least some of the relevant covariates due to the lack of publicly available metadata, which could have impacted the accuracy of our findings. A subset of these covariates could include the patient’s age, tumor size, clinical stage, ER and progesterone receptor status, and histologic subtype. In addition to these covariates, there are some questions that could not be answered because of the original study design that involved surgically collecting samples after treatment. As such, a direct analysis of samples collected before and after treatment is not possible.

We purposefully selected our cutoff criteria in the various analyses such that the statistical significance value was primarily used to determine the threshold. Our decision to not impose a fold-change cutoff enabled us to identify genes with relatively small but statistically significant changes in gene expression. This approach at least partially accounts for the cellular heterogeneity in bulk RNA-seq experiments [42,130,131]. We anticipate that adjusting our fixed threshold approach to a different set of cutoff values could alter the results.

Although our use of robust and established large-scale statistical methods should improve confidence in our results, the possibility of false-positives is inevitable. Consequently, we anticipate follow-up experiments in the wet lab to further validate our results. Additional studies that compare our results to those from triple-negative and/or ER- breast cancer patients could also be beneficial in efforts to anticipate precision medicine efforts.

## 5. Conclusions

Our analysis identified genes, pathways, and mechanistic transcriptional markers that could contribute to improved understanding, prognostics, and characterization of mechanisms for Letrozole resistance in ER+ breast cancer patients. Future work is needed to better characterize the identified gene products that do not display a well-understood function to better understand how the tumor microenvironment interacts with Letrozole. Further analysis of our predicted biomarkers could also yield clinical applications that enable treatment regimens specified to particular subsets of patients.

## Figures and Tables

**Table 1:** Metrics for the original 58 ER+ breast cancer samples treated with Letrozole.

Metrics	Study Information
Gene Expression Omnibus (GEO) Study Identifier	GSE145325
Title	RNA sequencing of ER+ breast tumor treated with Letrozole
Platform	Illumina HiSeq 3000
Diagnostic criteria	Resistance to estrogen suppression was defined by a preoperative endocrine prognostic index (PEPI) ≥ 4 and/or evidence of cancer relapse after a median follow-up of 5 years
Number of responders vs. non-responders	36 vs. 22

**Table 2:** Metrics and information for the top five statistically significant differentially expressed genes.

Gene Symbol	Log2 Fold-Change	Log Counts Per Million (CPM)	FDR *p*-Value	Gene Name
*SOX11*	3.87	3.74	8.07 × 10^−6^	SRY-box transcription factor 11
*S100A9*	4.53	5.06	1.77 × 10^−5^	S100 calcium-binding protein A9
*S100A8*	4.7	2.96	1.77 × 10^−5^	S100 calcium-binding protein A8
*IGLV3–25*	5.15	6.27	0.000184	Immunoglobulin lambda variable 3–25
*MMP7*	4.11	4.84	0.000665	Matrix metallopeptidase 7

**Table 3:** Metrics for all significantly affected intracellular signaling pathways and their predicted modulation.

Rank	Pathway Name	Total # of Pathway Members	# DEGs in Pathway	Bonferroni *p*-Value	Predicted Modulation
1	PLK1 signaling events/anti-tumoral activity	44	23	5.84 × 10^−10^	Activated
2	Syndecan-1-mediated signaling events/leukocyte adhesion	30	8	0.00018429	Activated
3	FOXM1 proliferation-associated transcription factor network	36	14	0.0105863	Activated
4	HIF-1-alpha transcription factor network/oxygen homeostasis	60	17	0.01923722	Inhibited

**Table 4:** Ranked list of top five repurposed therapeutic targets predicted to aid with the resistance phenotype.

Target Symbol	Target Name	Weighted Score (Higher Is Better)
VEGFA	Vascular endothelial growth factor A	2220.5
ESR1	Estrogen Receptor 1	1771
MMP9	Matrix Metallopeptidase 9	1699
FGFR3	Fibroblast Growth Factor Receptor 9	1589
AKT1	AKT serine/threonine kinase 1	1571

**Table 5:** Description and metrics for the top five predicted mechanistic transcriptional markers from all detected genes, ranked in decreasing order by gain.

Feature	Gain	Cover	Frequency	Antibody Available	Log2FC (FDR *p*-Value)	Location
PRDX4	0.032	0.027	0.023	Monoclonal	0.807 (0.0326)	Secretory granules; the ER; and exosomes
E2F8	0.031	0.025	0.021	Monoclonal	1.4 (0.266)	Cytosol and nucleus
IQGAP3	0.024	0.020	0.016	Monoclonal	1.2 (0.266)	Cytosol and plasma membrane
ATP6V1C2	0.021	0.018	0.015	Monoclonal	2.44 (0.0263)	Cytosol; lysosomes; and exosomes
CDCA8	0.017	0.014	0.011	Monoclonal	1.37 (0.101)	Cytosol and nucleus

## Data Availability

Data is contained within the article and Supplementary Materials.

## Supplementary Materials

The following supporting information can be downloaded at: https://www.mdpi.com/article/10.3390/cimb46070424/s1, Table S1: Excel download for differentially expressed gene information. Table S2: Excel download for therapeutic targets.
